# Social ecology of resilience and Sumud of Palestinians

**DOI:** 10.1177/1363459316677624

**Published:** 2017-02-08

**Authors:** Mohammad Marie, Ben Hannigan, Aled Jones

**Affiliations:** An-Najah National University, Palestine; Cardiff University, UK

**Keywords:** cultural context, Palestine, resilience, social ecology, Sumud

## Abstract

The aim of this article is to provide an overview of theoretical perspectives and practical research knowledge in relation to ‘resilience’, the resilience of Palestinians in particular and the related concept of ‘Sumud’. ‘Sumud’ is a Palestinian idea that is interwoven with ideas of personal and collective resilience and steadfastness. It is also a socio-political concept and refers to ways of surviving in the context of occupation, chronic adversity, lack of resources and limited infrastructure. The concept of ‘resilience’ has deep roots, going back at least to the 10th century when Arabic scholars suggested strategies to cope with life adversity. In Europe, research into resilience extends back to the 1800s. The understanding of resilience has developed over four overlapping waves. These focus on individual traits, protective factors, ecological assets and (in the current wave) social ecological factors. The current wave of resilience research focuses on the contribution of cultural contextualisation and is an approach that is discussed in this article, which draws on Arabic and English language literature located through a search of multiple databases (CINAHL, British Nursing Index, ASSIA, MEDLINE, PsycINFO and EMBASE). Findings suggest that ‘Sumud’ is linked to the surrounding cultural context and can be thought of as an innovative, social ecological, approach to promoting resilience. We show that resilience is a prerequisite to ‘Sumud’, meaning that the individual has to be resilient in order to stay and not to leave their place, position or community. We close by pressing the case for studies which investigate resilience especially in underdeveloped countries such as Palestine (occupied Palestinian territories), and which reveal how resilience is embedded in pre-existing cultural contexts.

## Background

According to the United Nations (2012), three quarters of Palestinians were displaced or fled as a result of the 1948 war when Israel was created. After the 1967 war between Arabic countries and Israel, additional numbers of Palestinians became displaced or began living under military occupation (UN, 2012). As a result, Palestinians have experienced ongoing collective punishment, trauma, suffering, social distress and political oppression (Giacaman et al., 2010). Palestinians have responded in a resilient way by creating innovative strategies to cope with occupation practices and prolonged adversity. For example, ‘Sumud’ culture is considered a main social ecological source of resilience among Palestinian adults (Marie, 2015). However, there is a near absence of studies that have examined resilience from a social ecological perspective within a Palestinian cultural context (Marie et al., 2017). This article discusses resilience from a social ecological view, and draws on the Palestinian experience to consider implications for civilians living in chronic conflict.

## Methodology

The literature reviewed in this article was gathered in the course of a larger doctoral study completed by the first author (M.M.). Our aim is to gain an overall view of theoretical perspectives and practical research knowledge in relation to ‘resilience’, the resilience of Palestinians in particular, and the related concept of ‘Sumud’. The following electronic databases were searched: CINAHL, British Nursing Index, ASSIA, Medline, PsycINFO and EMBASE. Keywords consisted of resili* AND Arab* OR Muslim*, resili* AND Palestin*, Sumud. These words in the Arabic language were also used to identify items indexed in Google Palestine, *An-Najah University Journal for Research* and Islamic University Gaza Journal of Research. Both books and articles about resilience were included. Items retrieved and used in the review included references relating to resilience and ‘Sumud’ of Palestinian, Arabic and Muslim adults. Articles that focused on hardiness and coping among children were excluded. In total, the search identified 222 items; duplicate items were removed, and a sub-set of the remaining is used in this article. The framework of Rees (2003) was consulted to help with appraisal and critique. This is particularly useful in the analysis of empirical articles, and the following aspects were considered: instrument, aim, sample, data collection, limitations or bias, key findings, ethical and procedural rigour. For empirical studies with statistical methods, reliability and validity were taken into consideration.

The article consists of several sections: the history of the concept of resilience, the social ecology of resilience, resilience in the Arabic and Islamic context, resilience among Palestinians and ‘Sumud’ as a social ecological idea. Finally, the relationship between resilience and ‘Sumud’ is discussed.

## History of the concept of resilience

European researchers started to use the concept of resilience in the 1800s (Jackson et al., 2007). The word itself comes from the Latin ‘resilire’ (defined as to rebuild/recoil) (Phaneuf, 2008). ‘Resilience’ also began to be used in psychiatric writing to describe children whose parents have a mental illness, live in adverse conditions and yet are invulnerable to mental illness. In this context, it appeared as a replacement for the word ‘invulnerability’ (Earvolino-Ramirez, 2007).

According to Ungar (2011, 2012), Ungar et al. (2007), Masten (2007) and others, there are four overlapping waves of resilience research:

1. Individual traits;
2. Protective mechanisms;
3. Developmental assets: individual and community;
4. Social ecological: culturally embedded understanding of resilience and ‘new voices’.

In the first wave, the initial conceptualisation of resilience focused on individual traits (Anthony, 1987; Garcia-Dia et al., 2013). According to the British paediatrician and psychoanalyst Bowlby (1969), the concept of resilience is based on attachment theory. He argued that the mother gives the infant a sense of security and self-confidence when she fulfils his or her basic needs. This protects the infant later on from life crises and helps him or her to cope with separation and adversity. However, it has also been noted that some individuals have the ability to become resilient in spite of lack of support from their families and communities (Turner, 2001).

In the second phase, resilience was conceptualised as a dynamic process and the interaction between genetic and environmental factors was considered (Rutter, 2012). The integration of genetic and other factors plays a role in developing resilience (Wu et al., 2013). The healthy individual uses internal defence mechanisms to cope (Phaneuf, 2008). Therefore, resilience can be a process of using internal and external protective factors to adapt to a situation (Garcia-Dia et al., 2013). Some researchers focus on resilience as a dynamic process of recovery or as a protective mechanism (Dyer and McGuiness, 1996; Garcia-Dia et al., 2013; Luthar et al., 2000).

Other scholars review the interaction between the environment and individual factors in determining resilience (Bosworth and Earthman, 2002; Cameron and Brownie, 2010; Humphreys, 2001; Ungar, 2005; van Kessel, 2013; Windle, 2011). The third wave of resilience conceptualisation is the shift to developmental assets, both individual and community. These scholars introduced a more ecological interpretation of resilience; they argued that resilience can be an outcome of interactions between individuals and their environments, and the progression which leads to these outcomes (Ungar, 2008). For example, resilience of the daughters of women victims of domestic violence can be affected by individual and environmental factors (Humphreys, 2001). In addition, children’s resilience in schools can be enhanced by focusing on individual and environmental factors (Bosworth and Earthman, 2002; Masten, 2001). However, as discussed below, there are still some limitations related to this understanding of resilience (Windle, 2011).

All three waves of resilience conceptualisation are open to critique. For example, culture also needs to be taken into consideration when we discuss resilience (Tusaie and Dyer, 2004). Resilience is sensitive to complex multidimensional interactions depending on context (Ungar, 2011). For instance, the resilient individual might be unable sustain their high resilience, every day, in each stage of his or her life. Other individuals may be resilient in spite of a lack of community or environmentally supportive resources. The individual who grew up with mentally ill parents or family will be considered vulnerable according to some of the above definitions. However, this is not always the case; these factors depend on the context and culture within which supportive factors interact. The child might live in a collective society where the extended family, neighbours and other supportive community resources buffer the challenges. In some situations, the surrounding culture supports the individual and helps him or her to use the available resources to their utmost (Ungar, 2011). Each individual has protective factors and risk factors in his or her surroundings; sometimes these factors can be converted from risky to protective and vice versa. It can vary from time to time and from person to person. For example, an individual can consider their parents as a protective factor that can be called on when needing to face adversity. Sometimes these same parents can become the risk factor and cause the adversity faced by the individual (Ungar, 2008). Cultural and contextual factors therefore affect the complex dynamic interactions between the sources of resilience (Ungar, 2011).

The fourth wave of understanding resilience has focused on the cultural context and other social ecological sources. Cultural values have been argued to play a crucial role in the collective resilience of the individual and community within a politically violent context (Sousa et al., 2013). According to Ungar (2008),

in the context of exposure to significant adversity, whether psychological, environmental, or both, resilience is both the capacity of individuals to navigate their way to health-sustaining resources, including opportunities to experience feelings of well-being, and a condition of the individual’s family, community and culture to provide these health resources and experiences in culturally meaningful ways. (p. 225)

This understanding is focused on the cultural and social ecological aspects of resilience, which the next section will discuss in detail.

## The social ecology of resilience

As identified above, an emerging direction for resilience research is the development and investigation of social ecological interventions (Ungar, 2011). Scholars who are the ‘new voices’ of resilience research include Ungar (2011, 2012), Ungar et al. (2007) and Masten (2007), among others. An important definition from Kent (2012) is that ‘resilience does not occur in isolation. It is an interactive process that requires something or someone to interact with. It is dependent upon context or environment, including our relationships’ (p. 111). It can be seen therefore that the individual brain is a responsive organ, interacting and operating within a social context, especially during adversities. For example, Supkoff et al. (2012) reported that children at psychological risk sometimes did surprisingly well. This was due to contextual factors which affected the developmental history of these resilient children.

According to Murray and Zautra (2012), community resilience is necessary for individual resilience in different cultures and contexts. The relationship between notions of ‘the individual’ and ‘the collective’ is key to understanding resilience as they also reported that shared identity, community collaboration and increased social ties promise to enhance the wellbeing of individuals under stress. A sense of collective strength underpinning individual strength was provided by Berliner et al. (2012) who argued that individuals struggling with personal challenges can support each other and become resilient. This will happen by strengthening community resilience, sustaining and revitalising culture through enhanced social networks and locally formulated values and resources. This also offers options for enhancing creativity and encourages shared activities.

This fourth and current wave of resilience conceptualisation understands resilience as culturally embedded. ‘New voice’ scholars suggest there are possible individual differences in resilience within the same context or culture. Some argue that sources of resilience may be different from culture to culture making it difficult to nominate fixed, ordered, global factors, especially as what determines protective and risk factors can differ significantly (Ungar et al., 2007). Although there may be some shared sources of resilience, the relative value of each and their origins can differ significantly from one culture to another. Each culture might provide meaning to a person living through adversity. For example, the effect of some risk factors on youths, such as political violence, depends on how they experience or deal with these risks within their cultural context (Ungar et al., 2007). The adaptive response of the individual to adverse circumstances is therefore determined through interaction between the individual and the context (Schoon, 2012).

The review of literature demonstrates that a number of scholars arrived at similar conclusions after conducting studies in non-Western cultures, including Palestine. Scheper-Hughes (2008) completed studies and worked in zones of political unrest including South Africa during the apartheid period, and Brazil. She tells us that the Western understanding of resilience is insufficient in other cultural contexts, especially in politically conflicted areas. In these places, there is an everyday form of resilience within oppressed and politically excluded communities. In overwhelming contexts, the ability to survive and exist is an important focus of resilience. The broader social and cultural context, family unity and sense of coherence which are often overlooked in Western understanding play crucial roles in understanding the resilience of people (Panter-Brick and Eggerman, 2012).

According to Barber (2013) in response to the Israeli occupation, similarly specific culturally different variables were identified as playing crucial roles in resilience. Contrary to the dominant expectation, the majority of Palestinian youths function effectively in spite of the surrounding risks. Due to the specific nature of the conflict, the youths have their own interpretation, exposure, participation and means of processing. Instead of imposing pre-existing understandings of resilience, there is instead a need to understand their ideology, their way of thinking and their created meaning regarding adversity. Gren (2009) carried out a study in the West Bank-Palestine to explore ways of maintaining everyday life in spite of adversity during the second Intifada including curfews, shelling and siege. Gren used interviews and fieldwork observations for over a year in one of the Palestinian camps. The study showed that people used tactics and practices of resilience to survive in the occupied territories. These included positive attitudes and strategies which were upheld by the surrounding cultural context. Steadfastness and the determination not to leave or be violently deposed from their land (‘Sumud’) was one of these strategies. Therefore, the resilience of the individual was discussed within a context of collective political resistance.

Resilience, therefore, has to be understood as being culturally embedded. It is a complex multidimensional interaction between the individual’s capacity and his or her physical and social ecologies. This understanding leads to a need to focus on how cultural context influences resilient individuals (Ungar, 2012). However, there is a near absence of studies which investigate resilience within conflict zones and in underdeveloped countries, and a lack of studies that investigate resilience within an Arabic or Muslim cultural context. A historical review of non-academic texts reveals that issues of resilience have long been considered and that much learning for the purpose of research can result from such a broader reading of literature.

## Resilience in Islamic history

The prophet Mohammad’s ‘PBUH’ (in Islamic culture, reference to Mohammad must be followed by the phrase ‘peace be upon him’ or ‘PBUH’) life story, with other examples of prophets in the Holy Qur’an, affected the way of thinking of believers in Islam. His life story (*Seerah*) was recognised as a good pathway or role model of resilience. Muslims try to follow Mohammad’s ‘PBUH’ *Sunnah* (*Sunnah* means all his behaviours and sayings in different life situations), which inspires followers to thrive or survive in difficult conditions (Ramadan, 2007).

Resilience was discussed when Al Balakhi suggested strategies to cope with life’s adversities during the 10th century. He suggested that the individual must be aware of themselves and their surrounding supportive resources. The human being can use his or her internal defence mechanisms known in Arabic as *AlhealAlnafsia* to cope with adversity in a positive manner. The individual can practise exercises or training to cope with small stressors but when he or she fails to cope, he or she could use external coping resources. This kind of training will help the individual to become more mentally flexible, gain experience, gain psychological strength and become more tolerant. This training, called at that time in Arabic *Altamaron Alnafsi*, meant that if the individual knows himself or herself to be vulnerable, he or she must try to avoid engaging in risks as much as possible to protect their mental health (Al Balakhi, AD 850–934).

## Resilience in Arab and Muslim communities

The concept of resilience is under-researched in the Arabic and Islamic regions. However, the studies below examined the concept within different contexts and countries. These studies were reviewed in the light of the social ecology of resilience and Ungar’s typology (Ungar et al., 2007). Abu Zahra (2004) carried out a qualitative study in the United States to explore the sources of resilience among immigrant Muslim women facing adversity after the events of 9/11. The mass media was distorting the realities of Islam and trying to alter the public image of Muslim identity. The sample reported that the main contributory sources of their resilience included their Islamic religion. Faith uniquely emerged as central, providing an underlying and broad support for these women. Their direct, close and positive relationship with Allah (God) was a significant source of support. In addition, the sample reported that collective supportive relationships inside and outside their families cultivated their resilience. Moreover, they emphasised the importance for resilience of the familial, gender, spiritual and personal protective process in Muslims’ cultural context, the women having grown up with adversity.

Beitin (2003) and Beitin and Allen (2005) carried out studies to explore sources of resilience among Arab American couples following the events of 9/11. They showed that the main sources were spiritual beliefs, resilient marriages and spousal support, and processes relating to identity and involving religion and nationality. Participants referred to their wider system, which included their children, relatives, worship practices and their relationship with the US Government and their governments back home. The participants had political/religious awareness which may have helped them to overcome crisis. This study also notes resilience resources within specific cultural contexts.

Milliano (2010) explored sources of resilience among 582 youths after a flooding disaster in Burkina Faso. Mixed methods were used, including interviews, focus groups and questionnaires. The objective was to explore what sources made people resilient during the disaster period. The findings suggest that tangible resources, such as food, and intangible resources such as social resources and Allah/God were helpful sources of resilience. This study showed that these resources strengthened or inhibited resilience among the youths who were influenced by their age, culture, context, gender and ethnicity. A dynamic interaction existed between the participants and their material resources. For example, male youths had more access to aid resources than females due to the surrounding culture and traditions.

In summary, cultural or contextual factors contribute to resilience. Social ecological influences play a significant role in resilience among Arabic or Muslim people. As we see in the above studies, male youths used available resources more than females due to factors associated with the cultural context (Milliano, 2010). Moreover, the immigrant women considered their cultural context which encourages the men to protect women and is significant for their resilience (Abu Zahra, 2004).

## Resilience among Palestinians

Few relevant studies on the resilience of Palestinians were found during the search conducted in support of this article. This is mostly attributable to the very limited government funding for health research (Mataria et al., 2009). Among the studies found, examples exist of investigations reflecting the third wave of resilience research which particularly takes into consideration developmental assets, both individual and in the community (e.g. Punamäki et al., 2001, 2011). Studies have also reflected social ecology ideas. A recent study explored mental health needs and sources of resilience and daily challenges that Palestinian community mental health nurses (CMHNs) face within and outside their demanding workplaces (Marie, 2015; Marie et al., 2016, 2017). An interpretive qualitative design was chosen to explore resilience and daily challenges. Totally, 15 face-to-face interviews were completed with participants. A total of 32 hours of observations of the day-to-day working environment and workplace routines were conducted in two communities’ mental health centres. Written documents relating to practical job-related policies were also collected from various workplaces. Thematic analysis was used across all data sources resulting in four main themes, which describe the challenges faced by CMHNs and their sources of resilience. These themes consist of the context of unrest, societal challenges, lack of resources and organisational challenges. Sources of resilience were identified as steadfastness, not leaving the land (‘Sumud’) and Islamic cultures, supportive relationships, making use of available resources, and personal capacity. The study concludes with a better understanding of resilience in Palestinian nurses which draws on wider cultural contexts and responses. Further studies of this type are needed, including in other parts of Palestine such as Gaza and East Jerusalem, and including participants from minority faiths and no faith at all.

Palestinian youth were included in an international resilience project, informed by social ecological ideas, led by Ungar (2008). This study’s sample consisted of 1500 youths from 14 different countries. Mixed methods were used, quantitative through questionnaires, and qualitative through face-to-face interviews. The sample included 114 Palestinian youths from both genders, with their ages ranging from 16 to 21. They were considered to be ‘coping well’ according to their behaviour in their cultural context. The youths explained that their experiences included facing adversity which made them resilient, especially when witnessing the clashes between Palestinians and soldiers, for example, when children threw stones in street protests. The study also reported that young people used resilience when faced with martyrdom, death and abuse and drug addiction. They talked of resilience resulting from expressing emotion, family support, involvement in youth clubs and sharing in building their local community. Moreover, the study found sources of resilience among youths may be similar but, significantly, the effort to promote resilience must always take into account that there are special considerations of each community’s cultural context. For example, the Islamic faith or spirituality is a more important factor among Palestinian youths than for young people in many other countries. Another relevant finding is that the Palestinian youths were significantly different from other youths, as they did not use ‘I’ when they mentioned their identity. They referred to the whole community when they tried to represent their identity. As Ungar (2008) observes, unlike the children in Palestine, children in Western countries rarely share in political activities. This observation raises methodological questions for resilience researchers, challenging approaches in which (for example) universal questionnaires are distributed to participants in places where resilience and its sources might vary remarkably

The findings of Al Ajarma’s (2010) study can largely be related to the up-to-date social ecological fourth wave of resilience research. This study found the following sources of resilience: education, family support, community network and social support and the arts. In addition, political awareness and activity helped individuals to find meaning in their lives and make sense of their struggles. Resources were discussed within the specific cultural context of the Palestinians who lived under occupation practices, political oppression, lack of security and lack of basic human rights. Participants talked about their previous experiences, and there is a need to investigate resilience based on the current experiences of Palestinian adults not all of whom are highly educated.

Makkawi (2012) undertook qualitative research to explore resilience among female students in the West Bank-occupied Palestine. Tape-recorded interviews were conducted with 15 participants, with data analysed thematically. Participants were newly graduated from high school in the 2005/2006 academic year, which was considered a significant academic challenge. They reported a number of resources which enabled them to achieve academically and to be resilient, including family support, the female-only school environment and supportive female teachers. Students also explained that specific personal characteristics such as self-esteem, self-confidence, internal locus of control, persistence and motivation to reach academic goals enabled them to succeed. This study showed that there was a need to support the students in a culturally sensitive way inside and outside the school. Gender segregation in schools might be an unwelcome idea in some cultures. Young women’s willingness to prove themselves and share in the public space was a significant resource within their cultural context.

Nguyen-Gillham et al. (2008) carried out a qualitative study to explore the resilience of Palestinian youth. The sample consisted of 321 Palestinians aged from 15 to 18 living in the 15 Ramallah areas. The sample was taken randomly from different schools and the researchers looked at how the adolescents interpreted and gave meaning to the concept of resilience in abnormal conditions. Sources of resilience were explored through separate focus groups conducted with both genders. Sources of resilience among boys were friends, families and sports activities, while for girls, they were found to include reading, writing and drawing, in addition to the pursuit of education. Some of the participants mentioned that living in their villages and cities was like living in a big prison due to prolonged movement restrictions. They tried to do specific or limited activities to promote their resilience based on what was possible or available in the context of the Israeli military occupation.

To summarise, for Palestinian youth, resilience is embedded in their capacity to conduct their lives as normally as they can in the face of a challenging context and lack of infrastructure resources. In this study, there are differences between sources of resilience among boys and girls; most girls develop their sources of resilience inside the homes. These findings might be due to the dominant conservative culture in Palestine. Most people believed that home is the most secure environment for girls due to the lack of security within the occupation context. The findings of normalising the abnormal are largely related to the ‘Sumud’ cultural context, the meaning of which is examined further below. How research participants shaped their pathway of resilience in an overwhelming context depended on what was culturally and contextually suitable and available. There were also differences between sources of resilience among males and females due to cultural context influences.

It is of note that there is sensitivity to cultural context in many of the studies reviewed above, linking them to the current social ecological wave of research into resilience. Resilience has also been discussed in relation to ‘Sumud’ culture within the Palestinian context (Gren, 2009; Isaac, 2011; Kårtveit, 2010; Taraki, 2008), a connection now explored in depth below.

## ‘Sumud’ culture as a social ecological idea

In 1978, the Palestinian Liberation Organization (PLO) recommended ‘Sumud’ as a way of helping people to remain steadfast in Palestine. Thus, ‘Sumud’ became a basic national concept and strategy for Palestinians in order to prevent the uprooting policy, to preserve identity and to restore dignity in the struggle for national liberty. In addition, Palestinians are deeply connected to their homeland, which is an integral part of their lives (Teeffelen, 2011). ‘Sumud’ is a very distinct, Palestinian, idea. It is the art of living to survive and thrive in the homeland in spite of hardship and under occupation practices. These skills of how to live inform all aspects of life, including the economic, political and social. They can also be used at many levels: individual, family and within the Palestinian community. ‘Sumud’ has been further divided into two types: tangible resources such as the infrastructure supporting basic needs (e.g. schools and hospitals), and intangible resources which include belief systems, religion and social and family support helping Palestinians cope with their chronic daily collective suffering (Teeffelen, 2011; Teeffelen et al., 2005). Both types of resource may help people with life’s challenges and be more resilient (Hobfoll et al., 2011). Differences also exist between passive or static ‘Sumud’, which focuses on the maintenance of life and homeland in the face of adversity, and dynamic ‘Sumud’ which emphasises occupation resistance (Teeffelen, 2009). Dynamic ‘Sumud’ is a form of personal and collective political defence or resistance against violent occupation practices (Meari, 2011).

According to Taraki (2008),

Resilience and steadfastness (Sumud) have been staples of the Palestinian ethos for generations now. Sumud’s incarnations have been many, but the dominant motif has been Palestinians’ determination to continue under adversity, fortified by their roots in their land, the strength of their traditions, and family and kin solidarity … A new conception of resilience has been taking root, one that is not based on an ascetic denial of frivolity, joy, or entertainment, but rather renders the very pursuit of happiness a manifestation of resilience and of resistance at the same time. The legendary resilience of Beirut’s [citizens], who are perceived as living life to its fullest despite the turmoil of war and strife is certainly an inspiration here. (p. 17)

Beirut is mentioned as it was the capital of Lebanon where Palestinian refugees demonstrated resistance to the Israeli occupation during the 1980s.

In his published book, *The Third Way*, Raja Shehadeh (1982: vii, cited by Wick, 2008: 336), who is a Palestinian author and human rights lawyer, writes of the everyday practices of ‘Sumud’:

Long before Arab politicians outside defined Sumud as a pan-Arab objective, it had been practiced by every man, woman and child here struggling on his or her own to learn to cope with, and resist, the pressures of living as a member of a conquered people. Sumud is watching your home turned into a prison. You, Samid^1^, choose to stay in that prison, because it is your home, and because you fear that if you leave, your jailer will not allow you to return … It is developing from an all-encompassing form of life into a form of resistance that unites the Palestinians living under Israeli occupation.

Teeffelen undertook face-to-face interviews with key Palestinians and asked them, ‘What is the meaning of the “Sumud” concept?’ There was a lack of consensus in defining it. Each tried to explain the concept from their own personal experience or perception. Each interviewee had struggled to survive and thrive on the land, overcome adversities and cope in their own way with the daily chronic challenges (Teeffelen, 2011). Palestinian artists have tried to explain the wide-ranging definitions of ‘Sumud’. One drew an old olive tree with deep roots; its branches try to be flexible in facing adverse conditions and strong winds. The tree is a symbol of peace, reward and history over thousands of years, and the roots are a symbol of steadfastness on holy land. The branches are a symbol of Palestinians’ resilience and the wind is a symbol of occupiers (Teeffelen et al., 2005).

The Palestinian culture is considered part of Islamic and Arabic culture, but the ‘Sumud’ concept is more significant to Palestinian culture alone and is deeply rooted in historical and religious contexts (Schiocchet, 2011). As a result of continuous adversities experienced by Palestinians, around one-third have needed mental health interventions (Afana et al., 2009). Leaving the homeland may be accompanied by even graver physical and mental health disorders. There are a wide range of coping mechanisms (Thabet et al., 2009) or adaptation strategies (Abu Elrub, 2005) embedded deeply in the ‘Sumud’ political and cultural context. Coping is inspired by the religious, political, cultural and historical ‘Sumud’ context (Albarawi, 2010). Palestinians have individual traits, such as hardiness, which are discussed as existing within ‘Sumud’ culture (Dokhan and Alhajjar, 2006; Hijazi and Abu Ghali, 2009). The prolonged history of occupation, displacement and siege motivated the Palestinians to develop their resilience strategies and gain experience in how to survive (El-Smairi, 2010).

The ‘Sumud’ concept is interwoven with the social ecological idea of resilience though the two are not the same; he who leaves the land may be quite resilient, but he is definitely not *Samid* (has no ‘Sumud’ status) (Teeffelen, 2011; Teeffelen et al., 2005). Therefore, resilience might be the prerequisite for ‘Sumud’. In the research context, there is a need to investigate resilience and ‘Sumud’ from a social ecological perspective. This includes in connection to the relationships between ‘Sumud’, resilience and health and wellbeing, an under-researched area in which new voices are beginning to make a contribution (Marie, 2015; Marie et al., 2017). Social ecological studies of resilience in under-researched cultural contexts or countries other than Palestine are also needed.

## Conclusion

Within Arabic and specifically Palestinian culture, resilience can be conceptualised as a prerequisite to understanding and achieving ‘Sumud’, meaning that the individual has to be resilient in order to remain steadfast in the face of daily challenges and not to leave their place or position. There is a gap in the literature investigating resilience especially in underdeveloped countries such as Palestine. However, there is an up-to-date wave of ‘new voices’ investigating resilience as embedded in the cultural context which may provide better understanding of experiences. It is important to bear in mind that there are unique pathways of resilience (Ungar et al., 2007), with qualitative research providing the means to understanding this (Ungar, 2003, 2004). In each cultural context, people are able to decide what are considered protective factors and what are considered dangerous. The relationship between risk and protective factors of resilience may be different from one place to another, meaning the researcher should not impose previous judgements or his or her preconceptions.

The idea of ‘Sumud’ might be transferable to other communities wishing to survive or flourish in circumstances of extreme hardship. Kirmayer et al. (2012) studied resilience among the indigenous aboriginal community in Canada. As with Palestinians, for these people, resilience was linked to connectedness to the land, cultural continuity, political activities, community solidarity and collective identity. They also have family and community connectedness and spirituality. Their language, history, traditions and storytelling are also considered to be sources of resilience. In terms of health, the dominant approach focuses on individuals, but this study and others show the need to attend to resilience features found in whole communities and to investigate their relationships to wellbeing. We conclude with the observation that resilience research needs to incorporate a consideration of socio-political and cultural contexts. We have revealed potentially important links between ‘Sumud’ and health-related resilience and in this article have started the work of connecting the two ideas together; however, this remains an under-researched relationship and more empirical studies, like those reported in Marie (2015) and Marie et al. (2017), are now needed. These include social ecological studies in Palestine, but also investigations in other parts of the world where connections between culture, context, resilience and health may prove important.

## Notes

1. The singular of ‘Sumud’ in the Arabic language is ‘Samid’.
